# Dendritic cell maturation in the corneal epithelium with onset of type 2 diabetes is associated with tumor necrosis factor receptor superfamily member 9

**DOI:** 10.1038/s41598-018-32410-5

**Published:** 2018-09-24

**Authors:** Neil S. Lagali, Reza A. Badian, Xu Liu, Tobias R. Feldreich, Johan Ärnlöv, Tor Paaske Utheim, Lars B. Dahlin, Olov Rolandsson

**Affiliations:** 1 Department of Ophthalmology, Institute for Clinical and Experimental Medicine, Linköping University, 58183 Linköping, Sweden; 2 Department of Ophthalmology, Sørlandet Hospital Arendal, Arendal, Norway; 3 National Centre for Optics, Vision and Eye Care, Faculty of Visual and Health Sciences, The University of South-Eastern Norway, Kongsberg, Norway; 4 Unit of Regenerative Medicine, Department of Medical Biochemistry, Oslo University Hospital, and University of Oslo, 0407 Oslo, Norway; 5 Øyelegesenteret i Tromsø, Fjærevegen 6, 9024 Tomasjord, Norway; 6 Department of Medical Sciences, Uppsala University, Uppsala, Sweden; 7 School of Health and Social Sciences, Dalarna University, Falun, Sweden; 8 Division of Family Medicine and Primary Care, Department of Neurobiology, Care Sciences and Society, Karolinska Institutet, Huddinge, Sweden; 9 Department of Translational Medicine - Hand Surgery, Lund University, Skåne University Hospital, 20502 Malmö, Sweden; 10 Department of Public Health and Clinical Medicine, Family Medicine, Umeå University, 90187 Umeå, Sweden

## Abstract

Type 2 diabetes mellitus is characterized by a low-grade inflammation; however, mechanisms leading to this inflammation in specific tissues are not well understood. The eye can be affected by diabetes; thus, we hypothesized that inflammatory changes in the eye may parallel the inflammation that develops with diabetes. Here, we developed a non-invasive means to monitor the status of inflammatory dendritic cell (DC) subsets in the corneal epithelium as a potential biomarker for the onset of inflammation in type 2 diabetes. In an age-matched cohort of 81 individuals with normal and impaired glucose tolerance and type 2 diabetes, DCs were quantified from wide-area maps of the corneal epithelial sub-basal plexus, obtained using clinical *in vivo* confocal microscopy (IVCM). With the onset of diabetes, the proportion of mature, antigen-presenting DCs increased and became organized in clusters. Out of 92 plasma proteins analysed in the cohort, tumor necrosis factor receptor super family member 9 (TNFRSF9) was associated with the observed maturation of DCs from an immature to mature antigen-presenting phenotype. A low-grade ocular surface inflammation observed in this study, where resident immature dendritic cells are transformed into mature antigen-presenting cells in the corneal epithelium, is a process putatively associated with TNFRSF9 signalling and may occur early in the development of type 2 diabetes. IVCM enables this process to be monitored non-invasively in the eye.

## Introduction

In recent years there has been a growing interest in the role of a low-grade inflammation in the pathogenesis of type 2 diabetes^1–4^, with numerous studies identifying circulating markers of inflammation, such as interleukin-6 and TNF-α, as predictors of the development of type 2 diabetes^5–7^. It has been suggested that type 2 diabetes is a pro-inflammatory cytokine-associated disease, where both the innate^7–9^ and adaptive^10,11^ immune system are involved. Among the complications of diabetes, ocular complications are often insidious and progressive. Diabetic retinopathy for example, is among the leading causes of blindness and has an underlying inflammatory component^12–14^.

Manifestations of diabetes in the eye, however, are not limited to retinopathy or macular edema but also extend to the cornea, where numerous studies have linked a corneal neuropathy to small-fiber diabetic peripheral neuropathy (DPN), primarily via the corneal sub-basal nerves as a putative ocular marker for the neuropathy^15,16^. In studies of the cornea in diabetes, *in vivo* confocal microscopy (IVCM) has provided a means for non-invasive clinical examination to assess the peripheral nerve^17^ and cellular^18,19^ status of the cornea as potential surrogate indicators of disease status and progression.

Besides monitoring of peripheral nerve degeneration, IVCM can also enable the monitoring of inflammatory activity in the cornea. Inflammatory cells in the cornea have been imaged and quantified using IVCM in cases of infection^20–23^, corneal transplantation^20,24^, contact lens wear^25,26^, dry eye disease^27^ and in diabetes mellitus^19^. These studies have used cell morphology in IVCM images to evaluate the density and maturity of dendritic cells (DCs) without the need for biopsy and with a high degree of correlation with immunostaining^28^. Three readily identifiable categories of cells in the corneal sub-basal nerve plexus have been noted: mature dendritic cells (mDCs), immature dendritic cells (imDCs) and globular cells^19,20,28^. Distinguishing between all three cell phenotypes (mature, immature, and globular) and performing analysis based on these cell subpopulations, however, has not to our knowledge previously been applied in a clinical study.

Given the strong association of inflammation and inflammatory cytokine markers with the development of type 2 diabetes and the further association of the eye with complications of diabetes such as retinopathy and DPN, we sought to investigate a possible relationship of corneal inflammatory dendritic cell subpopulations, distinguished by phenotype, with the development and progression of type 2 diabetes. Because of the possible confounding influence of local variations in the corneal DC subpopulations, we employed wide-area mosaicking of the corneal sub-basal plexus in three dimensions^29,30^ to depict and analyse cells within a large region of the central cornea. We further sought to investigate possible circulating inflammatory biomarkers associated with the corneal dendritic cells.

## Methods

### Study participants

One hundred and twenty-nine study participants, with and without type 2 diabetes mellitus, were initially part of a large population-based study conducted in northern Sweden in 2004^31^. For the purpose of this study in 2014, previous participants were invited as part of a 10 year follow-up. Details of the subject recruitment including inclusion and exclusion criteria and study flowchart are given elsewhere^30^. Subjects did not have active corneal disease or inflammation, and were not taking any topical ocular medications at the time of examination. Subjects did not report symptoms of dry eye, and review of patient records did not reveal any dry eye diagnoses or treatments. The performed examinations could not however, exclude the possibility of unreported or asymptomatic dry eye or ocular surface damage. Eighty-two subjects were recruited, and 163 eyes were examined. For the present study, data from 81 subjects with bilateral eye data were included. The cohort consisted of 39 individuals diagnosed with type 2 diabetes and 42 healthy individuals, not diagnosed with diabetes with either normal glucose tolerance (NGT, 33 individuals) or impaired glucose tolerance (IGT, 9 individuals). Those with diagnosed diabetes were further sub-grouped into short duration (diagnosis <10 years prior to ophthalmic examination, 11 individuals) and long duration (≥10 years since diagnosis, 28 individuals). Subgroupings were based on the oral glucose tolerance test (OGTT) and the clinical diagnosis of type 2 diabetes mellitus, with details given elsewhere^30^. The study subjects were all residents in the same county, located in the northern part of Sweden. The cohort was controlled in age (69.1 ± 1.2 years) in order to minimize any possible age-related effects. The study protocol was approved by the regional ethical review board in Umeå, Sweden (Ethical application no. 2013-21-31 M), and the study followed the tenets of the Declaration of Helsinki, with all participants giving written informed consent prior to inclusion.

### *In vivo* confocal microscopy of the cornea

As part of ophthalmic examinations in 2014, the central cornea in both eyes of all study subjects was imaged by *in vivo* confocal microscopy (IVCM) using a Heidelberg Retinal Tomograph 3 with Rostock cornea module (HRT3-RCM, Heidelberg Engineering, Heidelberg, Germany). Details of the imaging procedure have been described elsewhere^32^. Briefly, a drop of ophthalmic tear gel (Tear Gel carbomer 0.3%, Thea Pharmaceuticals, France) was used as coupling medium between the microscope objective lens and the corneal surface. The subject’s other eye fixated on a stationary white light to guide and stabilize the patient’s gaze. An adaptive imaging method was applied to create small axial image stacks (of 2–5 images, spaced at 1–2 µm depth intervals) at different depths through the sub-basal nerve plexus during the process of manual raster-scanning of the cornea laterally. The central cornea of all study subjects was scanned in this manner, with scans repeated for temporal, nasal, superior and inferior positions of the fixation target. As a prerequisite to achieve the best possible quality of nerve images for mosaic generation, the examiner aimed to maintain the sub-basal nerves in the best possible focus while scanning was performed by constantly adjusting the axial depth. The time required for examination of each eye varied from between five to ten minutes (mean of 32 examined eyes per day), and typically 1000 IVCM images were captured per eye.

### Image mosaicking and cell quantification

**Figure 1:**
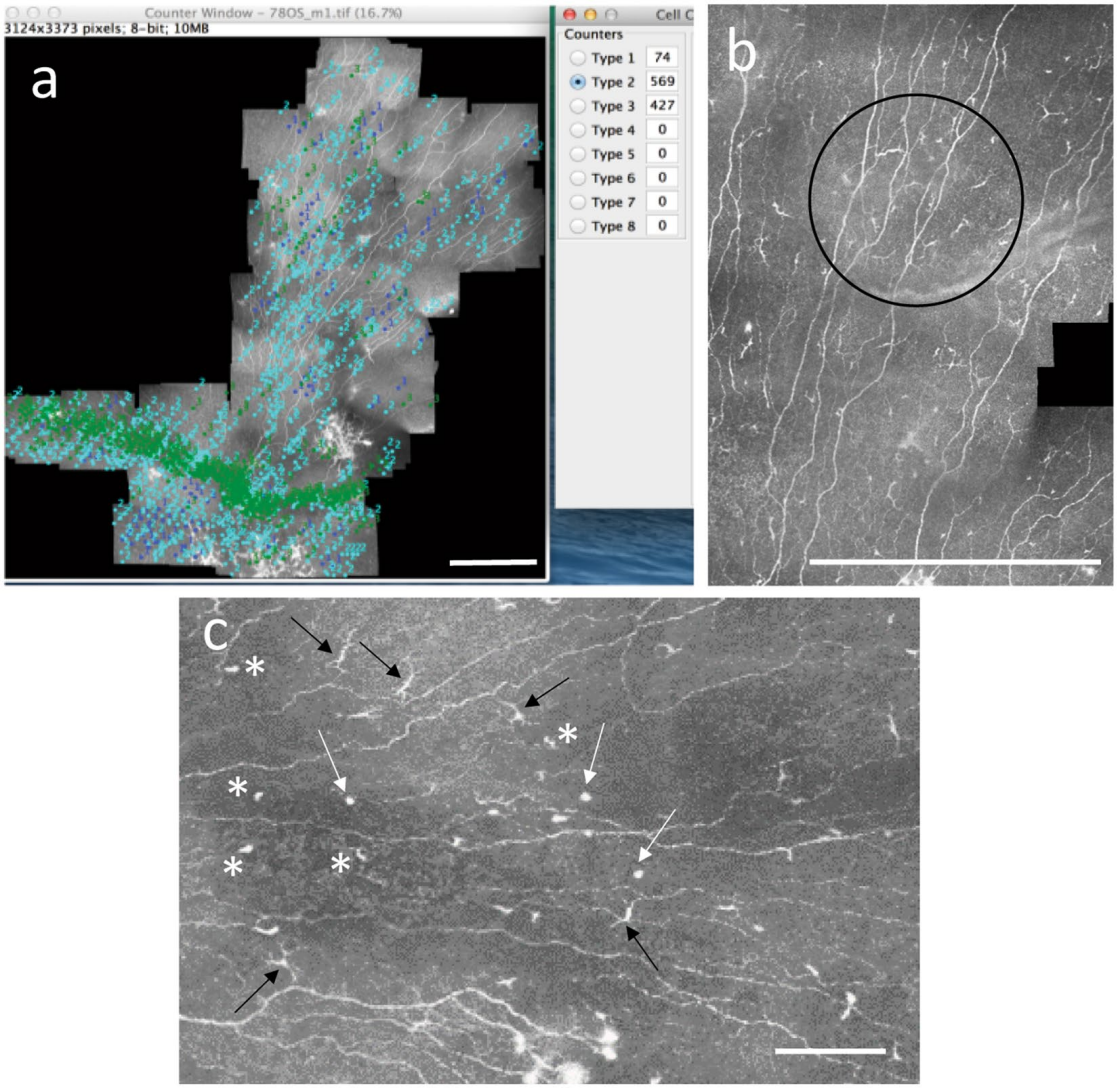
Quantification of cell parameters in the corneal sub-basal plexus. (**a**) Manual identification and quantification of different cell types in a mosaic image using ImageJ and the CellCounter plugin. The cells marked in dark blue (Type 1) are mDCs, those in light blue (Type 2) are imDCs, and those in green (Type 3) are globular cells. The mosaic is from a subject with normal glucose tolerance. (**b**) A zoomed-in section of a mosaic taken from a subject with long duration type 2 diabetes, indicating a clustering of mDCs (black circle). (**c**) Zoomed-in section of a mosaic indicating different morphologic appearance of the three different cell types quantified in this study. Black arrows indicate mDCs with clear, long dendrites, asterisks indicate imDCs with cell bodies or cell body with short dendrites visible. White arrows indicate globular cells, predominantly rounded and reflective and larger than imDC cell bodies, without visible dendrites. Note that for quantitative analyses, measurements were made independently by two trained observers and the average across both observers was taken. Scale bars A, B = 0.5 mm. Scale bar C = 0.1 mm.

Raw data from the IVCM examination were sorted to exclude images not containing the sub-basal plexus, and the plexus images served as input to an automated mosaicking algorithm that assembled the raw images taken in three dimensions into a two-dimensional mosaic with the maximal projection of sub-basal nerves^32^. One hundred sixty-two mosaics (bilateral eye data from 81 subjects) were assembled, with a mean mosaic assembly time of 7 minutes on a standard dual-core Windows PC. The mosaics represented a mean area of 5.95 ± 1.8 mm^2^ (range: 1.45–11.26 mm^2^) of the central corneal sub-basal nerve plexus just posterior to the corneal basal epithelium. Mosaics each consisted of an average of 522 individual input IVCM images taken at different depths of the sub-basal nerve plexus. All mosaics were analyzed for cell presence by two independent, trained observers, masked to the subject’s clinical status. Mosaics were viewed and analyzed using freely available ImageJ software^33^, and the CellCounter plugin (http://imagej.net/Cell_Counter). All cells were identified and counted manually in each mosaic by successive iterations of clicking on all cells of a given type, with each type indicated by a different number and color in the CellCounter plugin (Fig. 1a).

Three cell types were identified from mosaic images of the corneal sub-basal nerve plexus layer (Fig. 1b,c). Mature dendritic cells (mDCs) are antigen-presenting dendritic cells identified morphologically by a bright, reflective, slender cell body often having multiple long arm-like processes (dendrites) extending out from the main cell body, with a total end-to-end length exceeding 25 μm and often up to 45 μm or longer^19,20,28^. Immature dendritic cells (imDCs), MHC class II negative cells^34^, were identified as small, reflective cell bodies without discernible dendrites or short dendrites of total end-to-end length less than 25 µm. Globular cells resembled neither mDCs nor imDCs but instead were rounded or oval-shaped reflective cells, typically larger and more rounded than dendritic cell bodies, and without visible dendrites^20^.

Marked cells of each type were averaged across both observers, and divided by the mosaic area for each eye to determine the density of cells/mm^2^ in the sub-basal plexus. Additionally, the percentage proportion of each cell type relative to the total number of cells in the mosaic was calculated for each eye (averaged across both observers). The parameters of cell density and cell proportion were then each averaged across both eyes to yield single values for cell density and proportion per subject in the cohort to facilitate further analyses.

Besides cell density and proportion, several additional morphometric parameters were quantified for mDCs. These were the mDC size, total dendritic length, number of dendrites per cell, basoapical dendritic field area, and mDC clustering, the latter parameter as a further indicator of DC motility and activation^35,36^. These morphologic parameters were quantified for 10 randomly selected mDCs in the mosaic (where a total of <20 mDCs were present in the mosaic), or 20 randomly selected mDCs in the mosaic (where ≥20 mDCs were present in the mosaic). Kheirkhah *et al*.^27^ quantified DC size, number of dendrites and DC field in an earlier study; however, to this analysis we add the total dendritic length and clustering parameters. A more detailed description of the cell quantification methods and terminology used in this study is given in Supplementary Figs 1 and 2. The mDC size was defined as the two-dimensional area occupied by the mDC including the cell body and dendrites, measured using the thresholding function in ImageJ. Total dendritic length was defined as the maximum axial distance between the two outermost endpoints of dendrites, while basoapical dendritic field area of the mDC was defined as the area covered by a polygon that connects the endpoints of all dendrites; both parameters were measured in ImageJ. Finally, a mDC cluster was defined as ≥3 mDCs located in proximity to one another, with nearest dendrite endpoints located within a distance of less than or equal to twice the total dendritic length, as measured in ImageJ (Fig. 1b).

### Plasma protein multiplex inflammation panel

Useable venous blood samples were obtained from 78 of the 81 subjects examined, and consisted of samples drawn during the original study examinations in 2004 and again at the time of ophthalmic examinations in 2014. Samples were drawn in the morning after overnight fasting, and stored at −70 °C until the multiplex analysis was conducted in 2017. Analysis was conducted using the Olink Proseek Multiplex Inflammation I 96 × 96 kit (Olink Proteomics, Uppsala, Sweden) which is an oligonucleotide labelled, antibody-based protein detection and quantification assay. Each of the 78 plasma samples were distributed across 92 wells, each with a distinct human protein biomarker (entire list available at www.olink.com). In addition to the 92 wells, a negative control and three spiked controls (for IL-6, IL-8, and VEGF-A) were used to determine lower detection limits and to normalize measurements. The resulting values obtained were normalized protein expression levels that were log_2_-transformed to linear values proportional to protein concentration for subsequent analysis^37^.

For 2004 and 2014 samples, 24 and 25 proteins were excluded from analysis respectively, based on the criteria that at least 85% of participants had a valid measurement for the protein. For 2004 samples, excluded proteins were: BDNF, MCP3, GDNF, IL6, IL17C, IL17A, IL20RA, IL2RB, IL1α, IL2, TSLP, IL10RA, IL22RA1, IL24, IL13, ARTN, TNF, IL20, IL33, IFNγ, IL4, LIF, NRTN, and IL5. For 2014 samples, excluded proteins were: IL10RA, IL22RA1, IL24, IL13, ARTN, TNF, IL20, IL33, IFNγ, IL4, LIF, NRTN, IL5, BDNF, IL17C, IL17A, IL20RA, IL2RB, IL1α, IL2, TSLP, FGF5, MCP3, GDNF, and IL6. This left 68 and 67 proteins, respectively, for the analysis of 2004 and 2014 samples. Values below the lower limit of detection (LOD) were replaced with the value LOD/2. A previous validation study^38^ and the manufacturer (www.olink.com) gave the coefficients of variation for specific proteins.

### Statistical analysis

Relationships between cell parameters (density, proportion, and morphometric parameters) and the presence and duration of diabetes (across subgroups of NGT, IGT, short and long-term diabetes) were tested using the one-way ANOVA on ranks with Dunn’s method used to isolate the pairwise differences. In addition, values within groups of nondiabetes (NGT and IGT) and diabetes (regardless of duration) subjects were tested for differences by the Mann-Whitney test. Correlation between mDC proportion and DC cluster density was tested by Spearman’s rank correlation. A two-tailed P-value of <0.05 was considered significant, and analyses were performed using SigmaStat 3.0 statistical software (Systat Inc., Chigago, IL). Inter-observer differences in cell quantification were examined by the Bland-Altman method^39^ to determine the mean difference and 95% limits of agreement.

The parameters of mDC and imDC proportions were subsequently taken forward for analysis of associations with circulating plasma protein biomarkers from 2004 and 2014. Due to the relatively small cohort size relative to the number of biomarkers (close to unity ratio), it was determined that a strict Bonferroni multiple correlations correction would be too conservative and could potentially mask possible parameter associations. Instead, for discovery of associations between dendritic cell proportions and protein biomarkers we opted for performing separate linear regression models for each protein, adjusting for age, sex, and the presence of diabetes, since we aimed to detect associations independently of the possible modulating influences of these factors. For the linear regression models, a nominal value of P < 0.05 was considered significant. STATA Verison 14 was used for the analyses (StataCorp., College Station, TX). To evaluate the risk of type I error in the separate linear regression models, a false discovery rate of 0.25 was set and significant proteins from regression analysis were evaluated using the Benjamini-Hochberg procedure^40^ to identify candidate proteins associated with DC parameters.

## Results

### Mosaicking and DC quantification

One hundred sixty-two mosaics of the corneal sub-basal plexus obtained from 81 subjects were analyzed with a mean analysis time of 16.7 and 24.7 minutes per mosaic, respectively, for the two observers. Analysis time varied with the number and distribution of cells within the particular mosaic, ranging from 1 to 147 minutes.

The density and proportion of the three cell types within the sub-basal plexus were calculated and grouped according to stage of glucose metabolism. While no significant changes in cell density were found among the groups (Supplementary Table 1), the proportion of mDCs was elevated in type 2 diabetes (P = 0.013) relative to those without diabetes, and particularly in short-duration diabetes of <10 years relative to NGT (P = 0.028, Table 1). Post-hoc testing revealed a doubling in the proportion of mDCs (median 10.6%) in short duration diabetes relative to NGT (4.5%).

**Table 1:** Sub-basal plexus inflammatory cell proportion for three types of dendritic cells quantified in sub-basal plexus mosaics.

	Cell Type		
	mDC	imDC	Globular
NGT	4.5 (2.0, 7.5)	87.2 (68, 91)	5.7 (2.6, 28.4)
IGT	6.4 (3.9, 8.9)	86.4 (78, 92)	6.5 (1.0, 12.0)
T2DM < 10 y	10.6 (4.5, 17.9)	71.3 (66, 86)	8.5 (5.4, 18.8)
T2DM 10+	6.4 (4.3, 11.9)	82.7 (72, 88)	8.8 (4.3, 15.1)
ANOVA P	**0**.**028**^a^	0.45	0.65
Nondiabetes	4.9 (2.3, 8.1)	87.0 (68, 92)	5.8 (1.3, 26.7)
T2DM	7.2 (4.1, 12.8)	82.6 (71, 88)	8.5 (5.0, 16.5)
Mann-Whitney P	**0**.**013**	0.18	0.26

^a^One-way ANOVA with Dunn’s post-hoc method. Cell proportion given as median proportion (% of total cells) with 25^th^ and 75^th^ percentiles given in parentheses. T2DM = type 2 diabetes mellitus.

Of the morphometric parameters measured for mDCs, only clustering of mDCs was associated with the presence of diabetes (Table 2). Median cluster density was elevated in diabetes relative to those without diabetes (P = 0.045). Relative to glucose metabolism stage, mDC clustering followed a similar pattern as mDC proportion, with an early increase and reduction in the long-term, though this variation did not reach significance (P = 0.18). The cluster density, however, positively correlated with the proportion of mDCs in the sub-basal plexus (Spearman rho = 0.48, P < 0.001).

**Table 2:** Mature dendritic cell clustering in the sub-basal plexus.

	Median clusters/mm^2^	Q1, Q3
NGT	0.000	0.000, 0.188
IGT	0.076	0.021, 0.204
T2DM < 10 y	0.123	0.062, 0.255
T2DM 10+	0.109	0.000, 0.385
ANOVA P	0.18	
Nondiabetes	0.047	0.000, 0.189
T2DM	0.123	0.036, 0.303
Mann-Whitney P	**0**.**045**	

**Figure 2:**
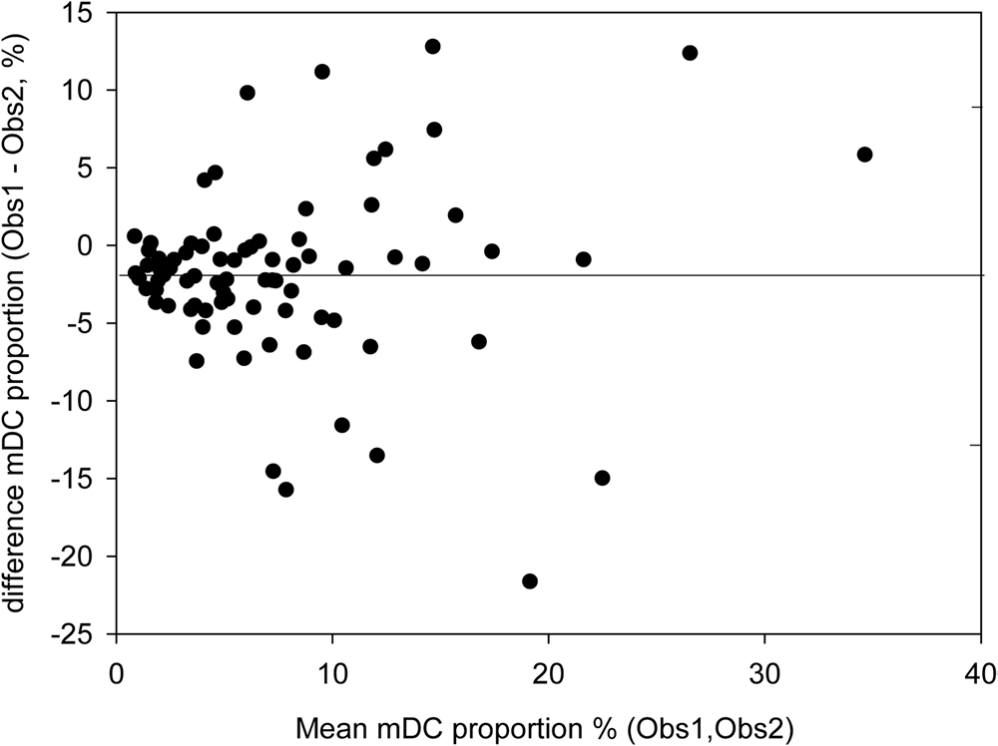
Bland-Altman analysis of inter-observer differences in cell quantification in the corneal sub-basal plexus. The mean difference in cell proportion between observers of 1.92% is indicated by the solid line, while dashed lines indicate the 95% limits of agreement of ±11% around the mean difference.

A mean inter-observer difference of 1.92% and 95% limits of agreement of ±11% was found for manual counting of the mDC proportion between the two independent observers across all subjects (Fig. 2). Averaging of values from both observers and across both eyes of a given subject served to reduce the inter-observer variability prior to further analyses.

### Association of cell parameters with blood plasma biomarkers

In multivariate regression models with mDC or imDC proportion as the dependent variable, only tumor necrosis factor receptor super family member 9 (TNFRSF9) was found to be associated with DCs (Table 3). The mDC proportion was positively associated with the protein concentration of TNFRSF9 in 2014 and in 2004. Conversely, TNFRSF9 was negatively associated with imDC proportion in 2014, but not in 2004. After further multiple comparisons correction, only the association of mDC proportion with TNFRSF9 from 2004 samples was significant; however, the protein remained top-ranked in 2014 samples for association with both mDC and imDC proportions.

**Table 3:** Associations of blood plasma proteins with mature and immature dendritic cell proportions in the cohort.

Cell type	Year	Protein	Adjusted for age and sex			Adjusted for diabetes presence		
			β	95% CI for β	P-value	β	95% CI for β	P-value
mDC	2014	**TNFRSF9**	**1**.**96**	**(0**.**52**, **3**.**40)**	**0**.**009**	**1**.**70**	**(0**.**33**, **3**.**07)**	**0**.**017**
		CCL4	1.59	(0.17, 3.00)	0.031	1.70	(0.39, 3.01)	0.013
		TGF-α	1.74	(0.32, 3.16)	0.019	1.46	(0.07, 2.85)	0.043
		β-NGF	1.51	(0.07, 2.96)	0.044	1.44	(0.07, 2.81)	0.044
	2004	**TNFRSF9**	**2**.**23**	**(0**.**87**, **3**.**60)**	**0**.**002**	**2**.**14**	**(0**.**83**, **3**.**45)**	**0**.**002**
imDC	2014	**TNFRSF9**	**−0**.**034**	**(−0**.**065**, **−0**.**002)**	**0**.**039**	**−0**.**033**	**(−0**.**063**, **−0**.**002)**	**0**.**039**
		IL-10Rβ	−0.034	(−0.065, −0.002)	0.039	−0.033	(−0.063, −0.002)	0.039
	2004	HGF	0.032	(0.001, 0.063)	0.049	0.036	(0.005, 0.067)	0.024

All proteins from the regression analyses with P < 0.05 are shown. β represents the regression coefficient, where a positive value indicates a direct relationship and a negative value indicates an inverse relationship. TNFRSF9 (in boldface) was the only protein associated with mDC proportion in both 2004 and 2014 blood samples, and was inversely related to imDC proportion in 2014. Applying Benjamini-Hochberg adjustment, only TNFRSF9 in 2004 samples was strictly associated with mDC proportion (P_crit_ = 0.0035).

### Cell distribution across the sub-basal plexus

**Figure 3:**
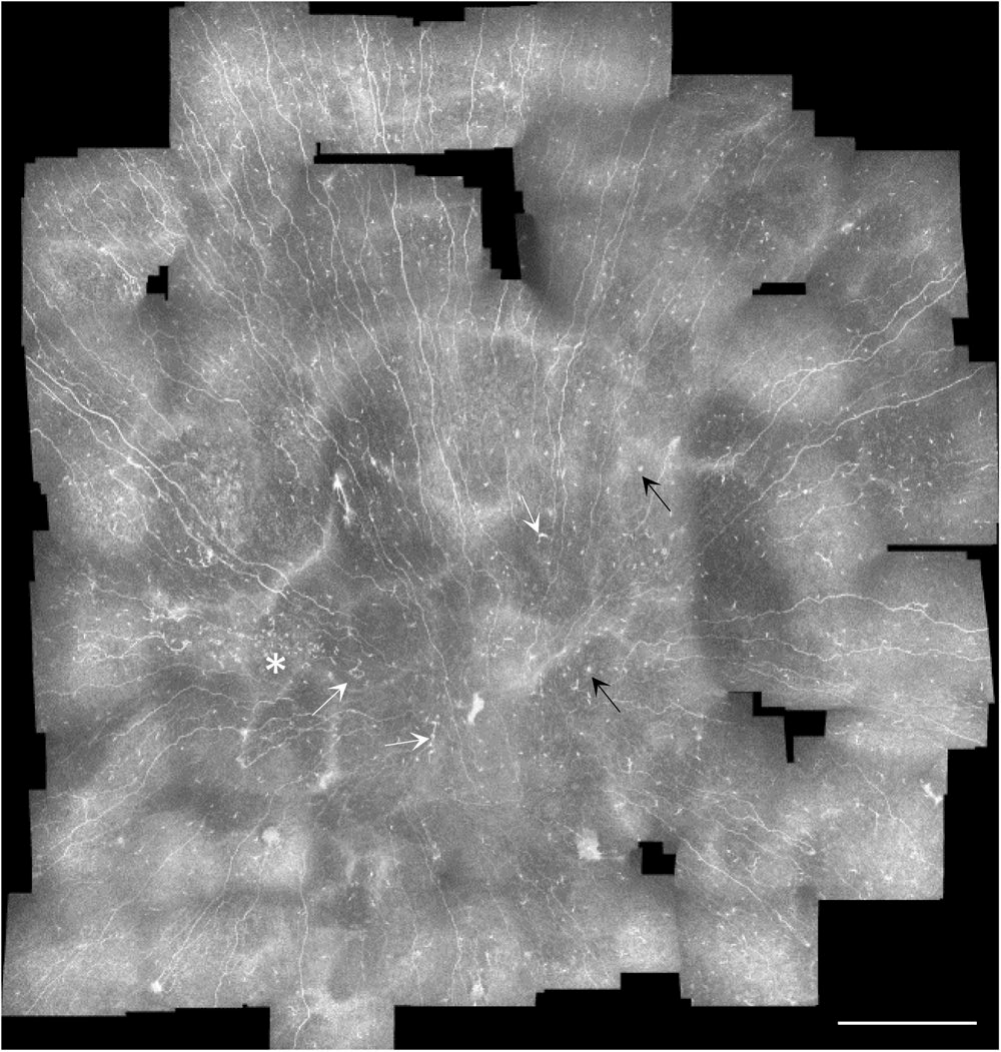
Sub-basal nerve plexus mosaic from the central cornea taken from the right eye of a subject with long-duration type 2 diabetes (12 years). White arrows indicate mature antigen-presenting dendritic cells, black arrows mark globular cells, and the asterisk shows a localized area with a high concentration of immature dendritic cells. Note the variable presence and density of cells depending on the specific local region of the plexus, which is typically analyzed using single image frames 400 × 400 µm in size. Scale bar = 0.5 mm. The entire set of original high-resolution images of sub-basal plexi from the cohort is freely available^32^.

**Figure 4:**
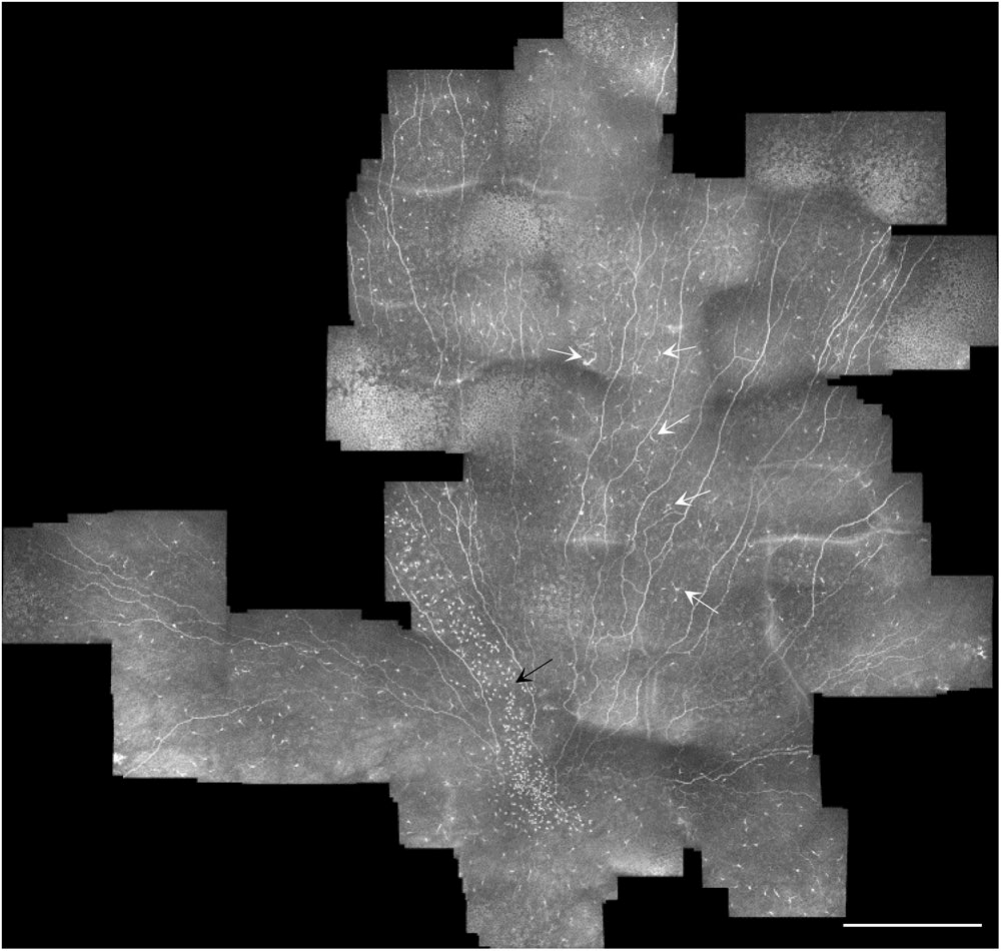
Sub-basal nerve plexus mosaic from the central cornea taken from the left eye of a subject with NGT. White arrows indicate mature antigen-presenting dendritic cells, while the black arrow marks a high density of globular and immature dendritic cells appearing in a pattern extending towards the corneal apex. Note the variable density and type of cells present in different regions of the plexus. Scale bar = 0.5 mm.

Besides enabling quantitative analyses of inflammatory cells, mosaic images of the corneal sub-basal plexus revealed the distribution of cells across the sub-basal plexus. In some cases, the plexus contained local regions of sparse and dense cell presence (Fig. 3). In other cases, cells in the plexus were distributed in a specific pattern (Fig. 4).

## Discussion

By comprehensive analysis of cellular subsets from wide-area three-dimensional mosaic projections of the corneal sub-basal nerve plexus, a major finding in this study was that the proportion of mature antigen-presenting dendritic cells in the central healthy cornea more than doubled with the onset of type 2 diabetes. This proportional increase in mDCs was accompanied by a proportional decrease in imDCs, suggesting a maturation of corneal imDCs into mature antigen-presenting cells as type 2 diabetes develops. The mDCs arranged in clusters of cells in close proximity to one another became more prevalent in diabetes, suggesting a mobilization of mDCs from an initially randomly distributed state in the healthy cornea into organized groupings of cells with impaired glucose metabolism, a phenomenon related to T-cell activity^36^. Dendritic cells (or Langerhans’ cells) have been studied in the human cornea previously, and found to vary in density according to age^41^, with contact lens wear^25^, in cases of corneal inflammation^20^ and in DPN^19^. Although differing morphology of DCs has been acknowledged in these prior studies, the three subsets have not previously been separately quantified. Moreover, typically 3 to 5 raw IVCM images are chosen for analysis of DC density in such studies, a method that would be sensitive to local variations in DC distribution. Indeed, the small changes in mDC proportion and organization detected in the present cohort, while significant, would be difficult to detect without the wide-area imaging technique used in this study.

In cases of overt corneal inflammation, the DC density in the central cornea can increase by a factor of ten or more^20^. The doubling in mDC proportion observed herein would correspond to a subclinical, low-grade inflammation. Interestingly, in streptozotocin- induced diabetes in mice, it was similarly shown that as diabetes developed, the corneal DC density increased 2 to 3-fold relative to controls, and that the DCs were mature MHC-class II positive cells residing in the sub-basal nerve plexus layer^42^.

An additional noteworthy finding in this study was the association of TNFRSF9 with the stage of maturation of dendritic cells in the cornea. TNFRSF9, also known as 4-1BB, CD137 or induced by lymphocyte activation (ILA)^43–45^, was associated with an increase in the proportion of mDCs and conversely associated with the proportion of imDCs. TNFRSF9 is known to be expressed on immune cells of hematopoietic lineage, primarily on activated T-cells, including CD4 helper T-cells, CD8 T-cells, activated natural killer (NK) cells, natural killer T (NKT) cells, regulatory T-cells, mast cells, and dendritic cells^46–48^. The primary ligand for the TNFRSF9/4-1BB/CD137 receptor is TNFSF9/4-1BBL/CD137L, which is expressed on professional antigen presenting cells (APCs) such as dendritic cells, monocytes, macrophages, and activated B-cells. The ligand TNFSF9 is not inherently expressed by immature DCs, but its expression was reported to be induced subsequent to activation of T-cells^49^. TNFRSF9 signaling has furthermore been shown to stimulate maturation of DCs, accompanied by an upregulation of costimulatory ligands that lead to increased survival of DCs, as well as increased production of inflammatory cytokines such as IL-6, IL-10, IL-12, IL-15 and IL-27^46,48,50–52^. This evidence suggests that a possible T-cell activation with onset of type 2 diabetes may induce the maturation of dendritic cells into an antigen-presenting phenotype in the corneal epithelium.

Obesity has also been shown to stimulate expression of MHC class II on adipocytes and an activation of CD4 + T-cells, causing adipose tissue inflammation^53^. In the cornea, in a mouse model of obesity, MHC class II-positive mDCs present in the corneal sub-basal plexus were increased threefold relative to controls^42^. It has also been reported that CD4 + T-cells are involved in the pathogenesis of obesity and insulin resistance^54^. Evidence also suggests that obese individuals have higher levels of TNFRSF9 proteins and transcripts in adipose tissue compared to non-obese individuals^55,56^. Studies have also indicated that the removal of TNFRSF9 reduced obesity-induced adipose inflammation and improved both insulin resistance and glucose tolerance^57,58^. TNFRSF9 signaling is furthermore linked with the production of several classical inflammatory markers of type 2 diabetes, such as IL-1β, IL-6, and TNFα^46^.

Notably, in our cohort, both the proportion of mDCs and clustering of these cells were low in healthy individuals, increased in IGT, peaked within the first 10 years after diagnosis of diabetes, and declined slightly in chronic diabetes. TNFRSF9 protein levels mirrored this pattern in both 2004 and 2014, suggesting a low-grade chronic inflammatory process that may persist for many years in the same individuals, eventually reaching a plateau or subsiding in advanced cases. Similarly, in a study of DPN, corneal DC density was elevated 2 to 3-fold in diabetes relative to healthy controls, with the greatest increase observed in mild neuropathy, but declining in moderate and severe cases^19^. This suggests that the low-grade inflammation apparent with the onset of type 2 diabetes and its complications may reach an equilibrium in the longer term. This hypothesis requires further study.

A limitation of this study was the small cohort size relative to the number of protein biomarkers assayed. To evaluate the probability of type I error in the regression analysis, we determined the likelihood of potential false discovery^40^. After adjustment, TNFRSF9 was significantly associated with mDCs based on 2004 blood samples; however, TNFRSF9 was additionally the highest ranked protein for mDCs and for imDCs (inverse relationship) in further independent analyses with 2014 samples. These results give confidence that TNFRSF9 is likely to be associated with the observed dendritic cell maturation in the corneal epithelium with the onset of type 2 diabetes. To our knowledge, this is the first clinical evidence linking TNFRSF9 signaling with type 2 diabetes; however, additional studies with larger populations are warranted to confirm the present findings.

Another limitation of the present study was the narrow focus on the relationship of systemic markers of inflammation and corneal dendritic cells. It is plausible that in other tissues and organs (eg., skin), dendritic cells are present and that these could additionally be evaluated for signs of early cell maturation. Other body fluids including ocular fluids in direct contact with the cornea, such as the tear film and aqueous humor, could also be analyzed for specific proteins such as TNFRSF9. Finally, a more detailed examination of dry eye signs and ocular surface/epithelial damage in type 2 diabetes in relation to inflammation and dendritic cell populations is warranted. Although we could not retrospectively perform such examinations and analyses in the present study, new prospective studies are recommended to investigate these further possible relationships.

This study demonstrates the possibility to monitor changes in specific DC subpopulations in the cornea as a potential early indicator of immune activation in diabetes. Further studies are required to confirm and extend the potential application of DC subpopulation analysis to other ocular pathologies. In particular, longitudinal study designs following at-risk individuals would be facilitated by the non-invasive nature of IVCM. Antigen presenting cell maturation in the cornea could be monitored in diabetes complications such as retinopathy, neuropathy, and nephropathy as a potential early biomarker of a low-grade immune activation. Given the ease of accessibility of the cornea for non-invasive clinical imaging of inflammatory cell subsets and the role of DCs in both innate and adaptive immunity, a biomarker of DC maturation could possibly aid in the future detection and monitoring of the inflammatory component of type 2 diabetes and its complications. Further refinement of the present methods, for example sampling an optimal number of single fields of the sub-basal plexus and development of algorithms for automated DC counting, could enable a more routine clinical analysis of DCs in future studies.

In conclusion, by non-invasive imaging of the eye we found that with the onset of type 2 diabetes, dendritic cells in the central corneal epithelium mature into an antigen-presenting phenotype and form cell clusters. This process was associated with increased circulating levels of the TNFRSF9 protein, a factor involved in dendritic cell maturation and inflammatory cytokine production.

## Electronic supplementary material

Supplementary Information

## Data Availability

The datasets generated during and/or analysed during the current study are available in the Figshare repository, and are described in detail elsewhere^32^.
