# Supplementary material for: Dendritic cell maturation in the corneal epithelium with onset of type 2 diabetes is associated with tumor necrosis factor receptor superfamily member 9
Source: Sci Rep. 2018 Sep 24;8:14248. doi: 10.1038/s41598-018-32410-5 (PMC6155153; doi:10.1038/s41598-018-32410-5)
Supplement: Supplementary file 1 — Supplementary Information [file 41598_2018_32410_MOESM1_ESM.docx]

Dendritic cell maturation in the corneal epithelium with onset of type 2 diabetes is associated with tumor necrosis factor receptor superfamily member 9

Neil S. Lagali, Reza A. Badian, Xu Liu, Tobias R. Feldreich, Johan Ärnlöv, Tor Paaske Utheim, Lars B. Dahlin, and Olov Rolandsson

Supplementary Information

The Supplementary Information consists of the following items:

**Supplementary Table 1**

**Supplementary Figure 1**

**Supplementary Figure 2**

**Supplementary Table 1**. Sub-basal plexus inflammatory cell density for three types of cells quantified in subbasal plexus mosaics.

|  | Cell density (cells/mm^2^), mean ± SD | | |
| --- | --- | --- | --- |
|  | mDC | imDC | globular |
| NGT | 4.5 ± 5.9 | 56.5 ± 42.3 | 7.6 ± 11.5 |
| IGT | 5.2 ± 3.9 | 85.0 ± 111.4 | 8.2 ± 17.2 |
| T2DM <10y | 4.7 ± 2.4 | 44.2 ± 54.2 | 4.5 ± 3.0 |
| T2DM ≥10y | 4.4 ± 4.5 | 55.0 ± 67.0 | 7.8 ± 18.9 |
| ANOVA P | 0.23 | 0.32 | 0.78 |
| Nondiabetes | 4.6 ± 5.5 | 62.6 ± 62.9 | 7.7 ± 12.7 |
| T2DM | 4.5 ± 4.0 | 52.0 ± 63.1 | 6.8 ± 16.1 |
| Mann-Whitney P | 0.49 | 0.09 | 0.42 |
|  |  |  |  |
|  |  |  |  |


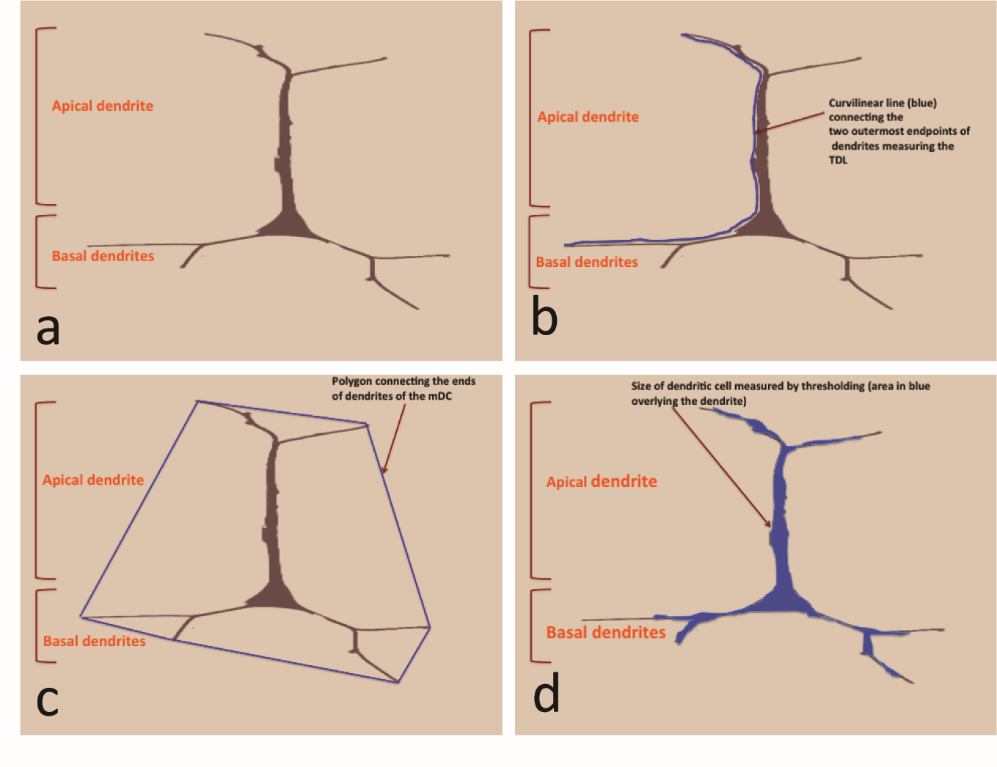


**Supplementary Figure 1**. Definition of several morphologic parameters of mature dendritic cells used for quantitative analysis. (A) A schematic illustration of a mature dendritic cell (mDC) with its apical and basal dendrites. The basal dendrite is located at the cell body (“soma”) end of the mDC, typically the thicker end of mDC. The apical dendrites are at the opposite end of the dendritic cell (terminal tuft). (B) The total dendritic length (TDL) is a drawn line connecting the two outermost endpoints of dendrites from apical to basal end (blue line). (C) Determination of the basoapical dendritic field area (BADFA) by drawing a polygon (in blue) connecting the ends of all dendrites of the mDC, and calculating the area of the polygon. (D) Schematic illustration of measuring the mDC size (area in blue). This is achieved using the thresholding function in ImageJ.^27^


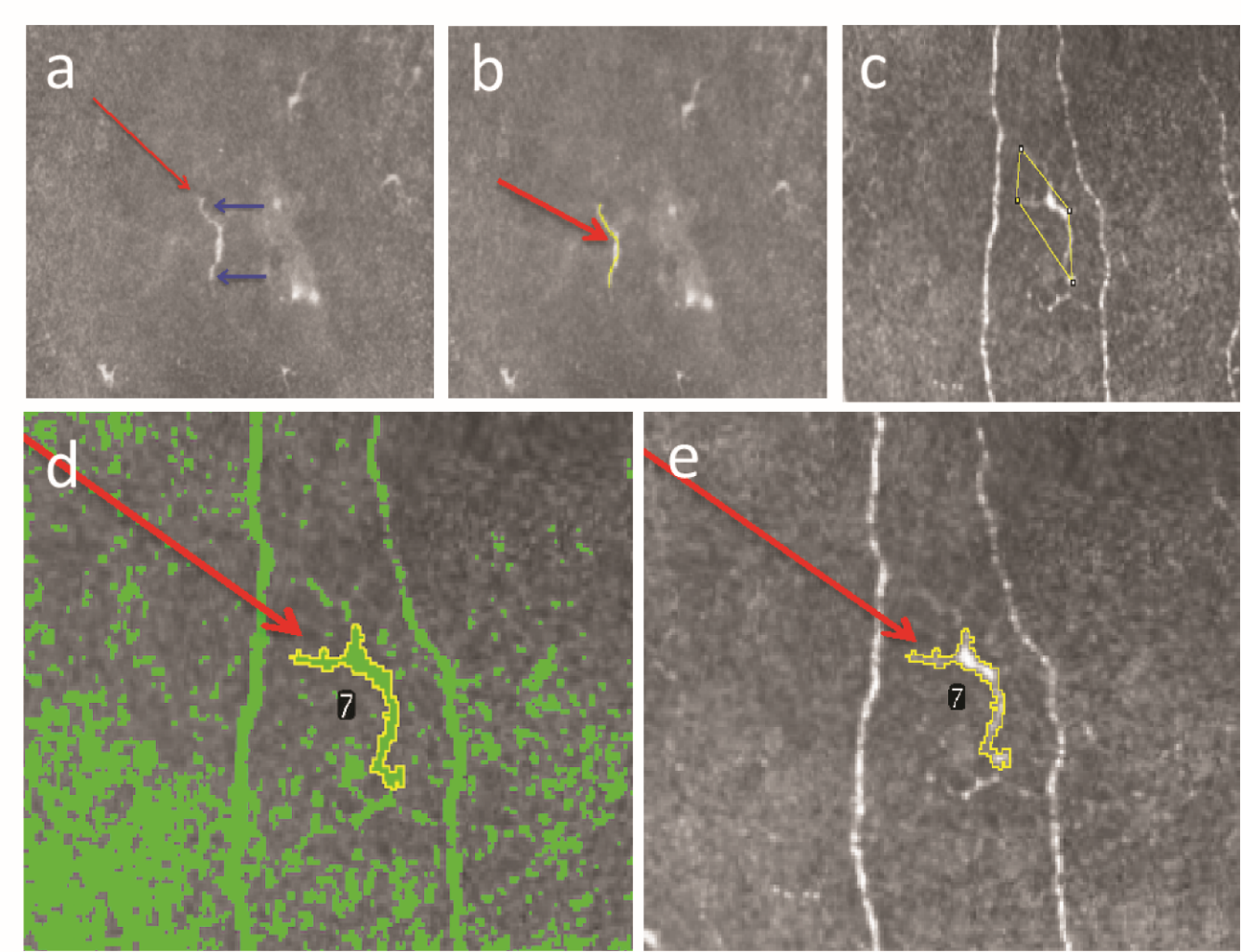


**Supplementary Figure 2**. Illustration of dendritic cell morphometric quantification from in vivo confocal microscopy images, using ImageJ. (A) Measuring the number of dendrites of a mature dendritic cell (mDC, red arrow) using image J. The number of dendrites is indicated by the blue arrows. (B) The total dendritic length (TDL) is measured by the length of the yellow curvilinear line, manually drawn. (C) The basoapical dendritic field area (BADFA) is measured using a polygon in ImageJ (yellow polygon), connecting the tips of all dendrites of the mDC. (D) Measuring the size of a mDC (red arrow) by using the thresholding function in ImageJ. The thresholded area is marked with a yellow boundary. (E) This yellow area is then used to calculate the dendritic cell size (number of pixels, or area contained within the boundary).
